# The therapeutic effects of dendrobium officinale polysaccharides on diabetes mellitus: from the perspective of gut microbiota

**DOI:** 10.3389/fendo.2025.1683752

**Published:** 2025-10-08

**Authors:** Jinyuan Wan, Ruihe Lin, Qiang Wu

**Affiliations:** ^1^ The State Key Laboratory of Mechanism and Quality of Chinese Medicine/Faculty of Chinese Medicine, Macau University of Science and Technology, Macao, Macao SAR, China; ^2^ The State Key Laboratory of Mechanism and Quality of Chinese Medicine, Macau University of Science and Technology, Macao, Macao SAR, China; ^3^ The Precision Regenerative Medicine Research Centre, Macau University of Science and Technology, Macao, Macao SAR, China

**Keywords:** *Dendrobium officinale* polysaccharides (DOPs), gut microbiota, diabetes mellitus, traditional Chinese medicine, immunomodulation

## Abstract

*Dendrobium officinale* is a traditional Chinese medicinal herb that has been extensively documented in classical medical texts for its effectiveness in treating diabetes mellitus. Modern pharmacological studies have shown that it possesses antitumor, antioxidant, immunomodulatory, and blood glucose- and lipid-lowering effects. *Dendrobium officinale* polysaccharides (DOPs), the main bioactive constituent of this herbal medicine, interact with the gut microbiota to reshape microbial composition, restore intestinal barrier integrity, modulate mucosal immunity, and ultimately ameliorate metabolic disorders. This review highlights the structural characteristics and bioactivities of DOPs, as well as the mechanisms by which gut microbiota are involved in the pathogenesis of diabetes mellitus. In particular, we point out that DOPs have significantly improved metabolic indicators related to diabetes by regulating intestinal microbiota. It aims to clarify the benefits of DOPs in ameliorating diabetes mellitus through gut microbiota modulation and provide new perspectives for its potential development as a prebiotic and for future clinical applications.

## 1 Introduction

Diabetes mellitus is a chronic metabolic disease featured by persistent hyperglycemia. Diabetes mellitus is primarily categorized as type 1 diabetes mellitus (T1DM), type 2 diabetes mellitus (T2DM), gestational diabetes mellitus(GDM), and other specific types (1, 2). In recent years, the incidence of diabetes mellitus has been increasing with a trend towards affecting younger people (3). According to the International Diabetes Federation (IDF), there will be approximately 589 million adult patients with diabetes mellitus (ages 20-79) worldwide in 2024, and up to 853 million patients can be expected by 2050 (4). Type 1 diabetes mellitus (T1DM) develops through an autoimmune attack on pancreatic β-cells, involving autoreactive CD4^+^ and CD8^+^ T lymphocytes, as well as activated macrophages (5). Genetic factors, such as HLA (DR3-DQ2/DR4-DQ8) and non-HLA genes (e.g., CTLA-4, PTPN22, VNTR) trigger autoimmunity and the formation of islet autoantibodies; Environmental factors, like Coxsackie B virus and gut dysbiosis-related decreased butyrate, contribute to β-cell stress and intestinal barrier disruption, leading to the translocation of bacteria/metabolites to pancreatic lymph nodes. Crossover antigens, such as viral or microbiota proteins, activate autoreactive CD4+/CD8+ T cells and macrophages through molecular mimicry, causing islet inflammation, β-cell destruction and lack of endogenous insulin secretion (5, 6).

The core mechanisms of T2DM involve insulin resistance and impaired β-cell function. Genetic predisposition and unhealthy lifestyle factors, such as chronic excess caloric intake and sedentary behavior, work together to reduce insulin sensitivity, downregulate GLP-1/GIP receptors, weaken the cAMP-PKA signal, and block the IRS1-PI3K-AKT and FOXO1 pathways. Additionally, the secretion of TNF-α and IL-6 increases, activating the IKKβ/JNK pathway, phosphorylating IRS1/2 (serine site), and blocking insulin signaling (7, 8). Gestational diabetes mellitus is the condition of abnormal glucose metabolism during pregnancy, marked by increased insulin resistance and insufficient relative insulin secretion (9). Gestational diabetes mellitus is typically diagnosed in mid to late pregnancy but can also happen in early pregnancy (10). Regardless of the subtype of diabetes mellitus, the common pathological outcome is persistent hyperglycemia and systemic metabolic dysregulation resulting from absolute or relative insulin deficiency (11).

The gut microbiota is the microbial community within the human intestinal tract and is the most extensive biological system within the body (12). It is important in maintaining host homeostasis (13). Increasing evidence suggests that gut dysbiosis is closely associated with the progression of diabetes mellitus. This association is mechanistically supported by microbiota-derived metabolites, modulation of intestinal barrier integrity, activation of inflammatory signaling networks, and coordination of immune responses. These factors collectively perturb the balance between insulin sensitivity and β-cell function (14, 15).

As a classical traditional Chinese medicinal herb, *D. officinale* exhibits broad pharmacological activities and has long been esteemed in traditional Chinese medicine for its capacity to benefit the stomach and promote fluid production, nourish yin and clear heat. Its principal bioactive constituent, DOP, is composed of 1,4-β-D-mannose, 1,4-β-D-glucose, and O-acetyl groups, conferring water solubility and non-starch characteristics (16). DOPs not only exhibit immunomodulatory, antioxidant, and antitumor activities but also possess hypoglycemic effects and can improve intestinal homeostasis (17). The International Scientific Association for Probiotics and Prebiotics (ISAPP) recently defined a prebiotic as “a substrate that is selectively utilized by host microorganisms conferring a health benefit” (18). DOPs exhibit resistance to digestion, thereby promoting the proliferation of beneficial gut bacteria. These bacteria ferment DOPs to produce short-chain fatty acids (SCFAs), which contribute to human health. These characteristics align with the definition of prebiotics. Therefore, elucidating the effects of DOPs on diabetes mellitus may hold significant clinical relevance. This review summarizes DOPs’ chemical structure, physicochemical properties, and bioactivities, while evaluating recent advancements in their ability to modulate gut microbiota and ameliorate diabetes. By elucidating underlying mechanisms and delineating prospective applications, we aim to provide novel insights and directions for the therapeutic exploitation of DOPs, either as a pharmacological agent or as a potential prebiotic, in the management of diabetes.

## 2 The structure and characteristics of *Dendrobium officinale* polysaccharides

*D. officinale* is an epiphytic herb of the genus *Dendrobium* in the Orchid family, which is native to East Asia and is predominantly distributed in the southern areas of China (19). In Traditional Chinese Medicine, *D. officinale* is believed to benefit stomach and promote fluid production while nourishing yin and clearing heat and is therefore prescribed for fluid impairment caused by febrile disease, dry mouth and polydipsia (20). The stems of *D. officinale* represent the principal medicinal part, rich in polysaccharides, phenanthrenes, flavonoids, alkaloids, and other bioactive metabolites (21, 22). DOPs have received considerable attention in recent years due to their multifaceted bioactivity, including immunomodulatory, antitumor, hypoglycemic, anti-inflammatory, and antioxidant effects (23, 24). DOPs are mainly composed of D-mannose and D-glucose linked by β-1,4 glycosidic bonds, and contain acetyl groups; the polysaccharide content is related to factors such as species, light, humidity, and temperature (19).

At present, various protocols have been developed for extracting DOPs, with the most used method being hot water extraction (HWE), cold-pressing (CP), freeze-thawing cold-pressing (FTCP), ultrasound-assisted hot water extraction (UHWE), microwave-assisted hot water extraction (MHWE), and enzyme-assisted hot water extraction (EHWE). Comparative studies have shown that FTCP provides the highest extraction yield and the most potent antioxidant activity, making it the preferred technique for DOPs recovery (25). Due to the different extraction efficiencies of these methods, the resulting polysaccharides vary in chemical composition, molecular weight, and macromolecular structure, all of which impact their bioactivities. The main structural features of some DOPs are summarized in **Table 1**. The biological activities and research methods of some DOPs are summarized in **Table 2**.

**Table 1:** Extraction methods and structural characteristics of some DOPs.

Name	extraction method	Mw (kDa)	Monosaccharide composition	Backbone	Branch	Reference
DOP	80°C hot water, 75% ethanol precipitation	4.56	Man: Glc: =5.78: 1.00	α-(1,3)-Glcp	β-(1,4)-Manp, α-(1,4)-Glcp	(26)
DOP-1	80°C distilled water, 80% ethanol precipitation	533.7	Man: Glc: Gal: Ara=3.13: 1.34: 0.02: 0.01	—	—	(27)
DOP-2	80°C distilled water, 80% ethanol precipitation	159.5	Man: Glc: Gal: Ara=3.13: 1.24: 0.12: 0.02	—	—	(27)
DOP-W3-b	100°C deionized water extraction, 80% ethanol precipitation	15.43	Man: Glc=4.5: 1.0	β-(1→4)-D-Manp, β-(1→4)-D-Glcp, β-(1→3,6)-D-Manp	β-(1→4)-D-Manp, β-(1→4)-D-Glcp, terminal β-D-Glcp, and O-acetyl groups attached to O-2 of β-(1→4)-D-Manp	(28)
DOP-1-A1	100°C distilled water and 1% polyvinylpyrrolidone extraction, 60% alcohol precipitation	130	Man: Glc: Ara=40.2: 8.4: 1	(1→4)-linked β-D-Manp, (1→4)-linked β-D-Glcp	(1→3) -linked Manp, (1→3) -linked Glcp	(29)
DOP-1-1	90°C hot deionized water, 80% ethanol precipitation	179	Man: Glc=5.9: 1.0	(1→4)-β-D-Manp, (1→4)-β-D-Glcp	—	(30)
DOPA-1	Extracted thrice with water and precipitated with anhydrous ethanol	394	Man: Glc=5.8: 1.0	1,4-linked Manp, 1,4-linked Glcp	1,3,4-linked Manp, 1,2,4-linked Manp, 1,4,6-linked Manp, 1,4,6-linked Glcp	(31)
DOPA-2	Extracted thrice with water and precipitated with 80% ethanol	362	Man: Glc=4.5: 1.0	1,4-linked Manp, 1,4-linked Glcp	1,3,4-linked Manp, 1,2,4-linked Manp, 1,4,6-linked Manp, 1,4,6-linked Glcp, 1,6-linked Manp, 1,3,4-linked Glcp	(31)
DOP	Ultrasound-assisted enzymatic extraction	2200	Man: Glc: Gal: Ara=74.17: 47.80: 9.03: 1.00	—	—	(32)
DOPa	100°C deionized water extraction, 71.25% ethanol precipitation	810	Man: Glc=5.6: 1.0	β-(1→4)-D-Manp, β-(1→4)-D-Glcp	residues	(33)
DOPb	100°C deionized water extraction, 71.25% ethanol precipitation	670	Man: Glc=5.9: 1.0	β-(1→4)-D-Manp, β-(1→4)-D-Glcp	residues	(33)

**Table 2:** Biological activities and research methods of some DOPs.

Bioactivities	Name	Purity	Study type	Model	Evaluation metric	Health outcome	Reference
Immuno-enhancing	DOP	Crude	*In vitro*	RAW264.7 macrophages BALB/c mouse spleen cells YAC-1cells	OD490nm Splenocyte proliferative index(SPI) TNF-α, IL-1β, IL-2, IL-4	Induce macrophage activation Induced T cell activation	(27)
	DOP-1	Purified					
	DOP-2	Purified					
Immuno-enhancing	DOP	Purified	*In vivo*	ICR mice induced by cyclophosphamide	Phagocytosis rate and phagocytic index of macrophages Blood routine examination IgA, IgG, IL-1, IL-6, IL-10 CD4^+^, CD8^+^	Enhance macrophage phagocytosis Increased anti-inflammatory cytokines	(32)
Enhance intestinal immune activity	DOP-W3-b	Purified	*In vivo*	ICR mice	INF-γ, IL-4, sIgA Ileal section	Enhance intestinal immune activity	(28)
Beneficial effects on intestinal microbiota	DOP	Crude	*In vitro*	*In vitro* fermentation model	SCFA DNA extraction and 16S rRNA gene sequencing	Digested by human intestinal microbiota during 48h of fermentation SCFA production increases	(34)
Antidiabetic effects	WDOE	Crude	*In vivo*	STZ/HFD induced T2DM model in C57BL/6J mice	FBG, FINS, HOMA-IR, HbA1c, OGTT, IPITT,TC, TG, LDL-C, HDL-G DNA extraction and 16S rRNA gene sequencing	Improve metabolism, regulate blood lipids, and reverse dysbiosis of the microbiota	(35)
	WDOP1	Purified					
Attenuates diabetic cardiomyopathy	DOE	Crude	*In vivo*	STZ induced diabetes model in Kunming mice	FBG, TC, TG, CK, LDH, MDA, T-SOD NF-Κb, TNF-α, IL-1β TGF-β1, Collagen I, Fibronectin	Lowering blood glucose and lipid levels Inhibiting oxidative stress, lipid accumulation, inflammatory responses, and myocardial fibrosis	(36)
Anti-cancer	DOPS	Purified	*In vivo*	AOM/DSS induced colitis associated colorectal cancer model in BALB/c mice	Clinical symptoms, intestinal permeability Inflammation score, number and size of tumors TNF-α, IL-1β, IL-10	Relief of chronic colitis Inhibit tumor growth Reduce intestinal permeability	(37)

Sun et al. (26) isolated a 4.56-kDa low-molecular-weight glucomannan (DOPs) from the 75% ethanol supernatant of *D. officinale*. These DOPs had a mannose-to-glucose molar ratio of 5.78:1.00, an α-(1,3)-Glcp main chain and α-(1,4)-Glcp plus β-(1,4)-Manp branches. It showed dose-dependent reversal of CTX-induced immune suppression and oxidative damage in mice by increasing immune organ indices, enhancing immune cell activity, and up-regulating IL-2, IFN-γ, TNF-α, SOD, and GSH-Px levels. Wang et al. (38) obtained DOPS-1 (1,530 kDa) from D. officinale stems using conventional hot-water extraction. This polysaccharide had mannose, glucose, and galacturonic acid ratio of 3.2: 1.3: 1 and contained (1→4)-β-D-Glcp, (1→4)-β-D-Manp, and 2-O-acetyl-(1→4)-β-D-Manp residues, giving it both antioxidant and antitumor properties. Studies have shown that the high mannose content of DOPs is linked to its pancreatic lipase-inhibitory activity, the degree of branching is associated with α-amylase inhibition, and the branched structure contributes to intestinal immunomodulatory effects (28, 39). Xie et al. (28) extracted *D. officinale* stem powder with 80% ethanol, followed by sequential elution with water and NaCl solution to yield the neutral fraction DOP-W. The activity of DOP-W surpassed that of the acidic fraction DOP-S. Subsequent precipitation and purification identified the most potent subfraction as DOP-W3-b, which exhibited a molecular weight of 1.543 × 10^4^ Da, a mannose-to-glucose molar ratio of 4.5:1.0, and a pronounced enhancement of intestinal immune responses in mice. Its immunomodulatory activity significantly exceeded that of W3-a (+18.9%) and W3-c (+53.5%). The drying protocol significantly affects the polysaccharide content of *D. officinale*. Freeze-drying is optimal for preserving the glucomannan-rich DOPs fraction (40). Collectively, these diverse DOPs extraction methods may lead to different compositions of DOPs and varied bioactivities.

## 3 Relationship between gut microbiota and diabetes mellitus

### 3.1 Composition and functions of gut microbiota

The human gastrointestinal tract houses most host-associated microorganisms, whose combined biomass exceeds that of human somatic cells by approximately one order of magnitude (41). The gut microbiota is made up of distinct bacterial phyla dominated by *Firmicutes*, *Bacteroidetes*, *Proteobacteria*, and *Actinobacteria*, which respectively make up 64%, 23%, 8%, and 3% of the community (42, 43). Different microbial taxa have specific functions and can be categorized as beneficial, neutral, and harmful bacteria based on their impact on human health. Early infancy is a critical period for establishing microbiota, during which microbial diversity increases rapidly and later stabilizes due to the combined effects of host genetics and environmental factors (12). Under normal conditions, the gut microbiota maintains a relatively stable equilibrium which is essential for human health; however, its dysbiosis can result in the development of various diseases, including obesity, allergic diseases, central nervous system disorders, and diabetes mellitus (44, 45).

### 3.2 Alterations of the gut microbiota in diabetes mellitus

**Figure 1:**
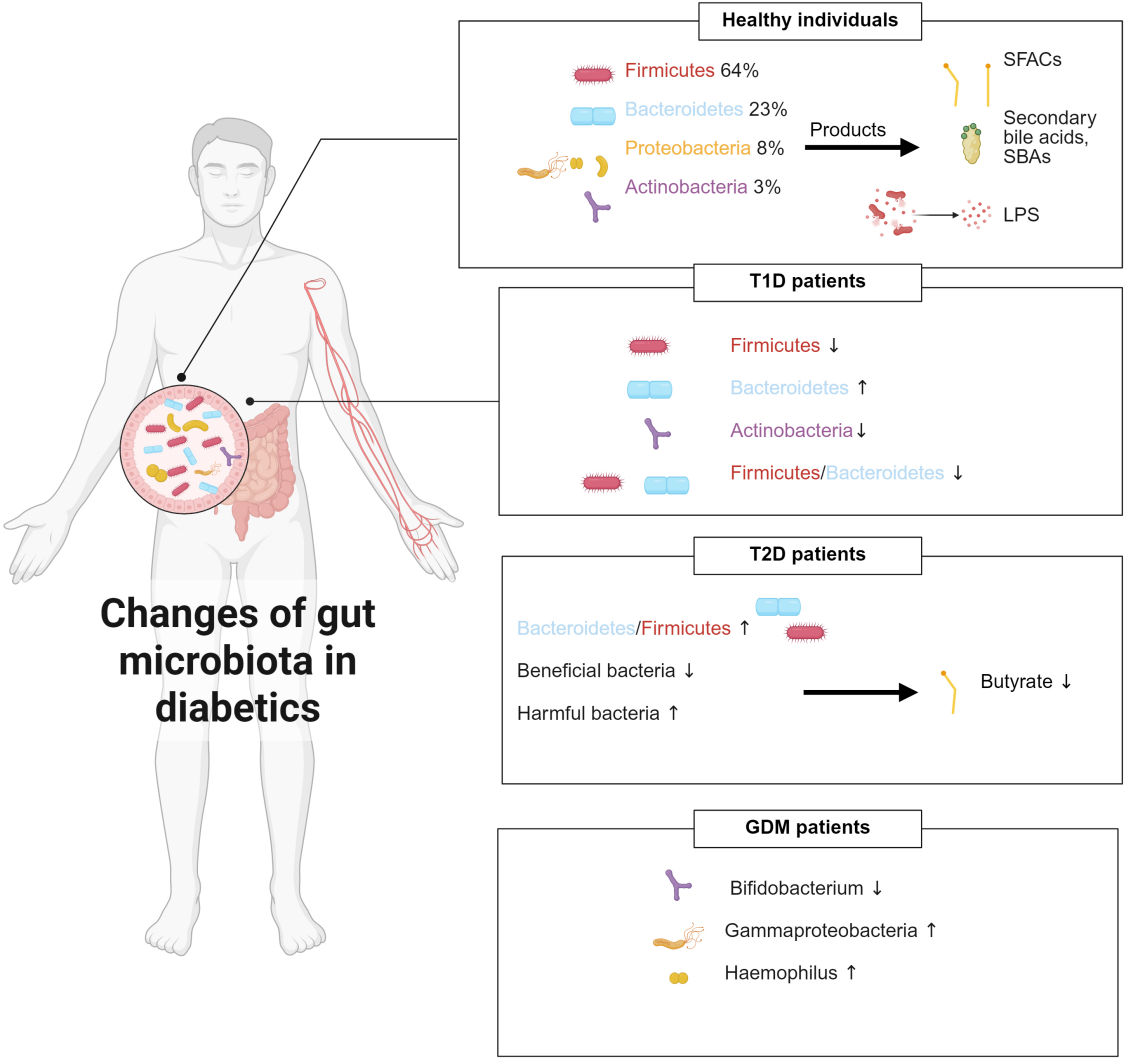
Gut microbiota composition changes under different physiological conditions and its dysbiotic shifts in diabetes mellitus.

Accumulating evidence consistently demonstrates that the composition of gut microbiota is significantly imbalanced in individuals with diabetes mellitus (**Figure 1**). Larsen et al. (46) were the first to show significant compositional differences between the intestinal microbiota of diabetic patients and healthy people. Specifically, the *Bacteroidetes*/*Firmicutes* ratio was significantly higher in the diabetic cohort and correlated positively with plasma glucose levels. Additionally, the relative abundance of the *Clostridia* class was dramatically reduced, while the *Betaproteobacteria* class was significantly enriched and also positively correlated with blood glucose levels (46). Qin et al. (47) identified around 60,000 markers associated with type 2 diabetes mellitus (T2DM) by conducting deep sequencing of gut microbial DNA from 345 Chinese individuals, including T2DM patients and non-diabetic controls. They revealed a decrease in beneficial bacteria and an increase in harmful bacteria in the T2DM gut by comparing the prevalence of genetic and functional markers between T2DM patients and non-diabetic controls. At the functional level, genes involved in membrane transport, oxidative stress resistance, and xenobiotic tolerance were enriched, while those related to bacterial motility, cofactor and vitamin metabolism, and butyrate biosynthesis were diminished (47). Murri et al. (48) provided the first evidence linking T1DM to alterations in the gut microbiome. Children with T1DM showed a significant reduction in *Actinobacteria* and *Firmicutes*, along with a notable increase in *Bacteroidetes*, resulting in a markedly decreased *Firmicutes*/*Bacteroidetes* ratio compared to healthy peers. Gestational diabetes mellitus (GDM) is also accompanied by distinct microbial shifts. *Bifidobacterium* is less abundant in GDM patients than that in normoglycemic pregnant women (49). In pregnant women with gestational diabetes, the abundance of the *Gammaproteobacteria* class and its associated genus *Haemophilus* is significantly increased, which is correlated with elevated levels of C-Reactive Protein (50). Interestingly, some studies suggest that despite the significant alterations in gut microbiota and the increased inter-individual variability observed during late pregnancy, with overall increases in *Proteobacteria* and *Actinobacteria* that can induce symptoms resembling metabolic syndrome, these changes are beneficial in the context of pregnancy, as they help provide energy for the fetus and prepare for lactation (51).

### 3.3 Gut microbiota metabolism and diabetes mellitus: underlying mechanisms

The gut microbiota and its metabolites are crucial in the onset and development of diabetes mellitus. These microbial communities help the host acquire the nutrients and facilitate the breakdown and fermentation of dietary substrates, which in turn affects carbohydrate metabolism and produces short-chain fatty acids (SCFAs), secondary bile acids, lipopolysaccharides, and other bioactive molecules (52). These metabolites interact with various host physiological systems through different pathways, influencing metabolic, immunological, and neurological functions (53–55). Hence, gut microbiota can be a therapeutic target for metabolic disorders. Interventions such as prebiotic or probiotic supplementation and fecal microbiota transplantation are currently being studied (56, 57). While the exact causal relationships and mechanisms linking specific microbial taxa or metabolites to diabetes mellitus are not yet fully understood, a significant amount of research indicates that gut microbiome signatures can serve as reliable biomarkers strongly linked to the development and progression of the disease.

Short-chain fatty acids (SCFAs)—primarily acetate, propionate, and butyrate—constitute the predominant metabolic products of the gut microbiota (58). Propionate enhances hepatic glycogen storage, upregulates lipoprotein lipase (LPL) activity, and attenuates the proinflammatory cytokine interleukin-8 (IL-8), thereby preserving intestinal barrier integrity (59). Furthermore, propionate exerts direct effects on pancreatic β-cells by stimulating insulin secretion and inhibiting β-cell apoptosis via the FFAR2–Gq–PKC signaling axis (60). Butyrate is avidly absorbed by colonic epithelial cells and serves as their principal energetic substrate, providing approximately 60–70% of their energy requirements (61). Acetate, which is present at higher concentrations in the colon than butyrate, is predominantly associated with *de novo* lipogenesis (61, 62). Beyond their role as metabolic fuels, SCFAs modulate host physiology through two principal signaling mechanisms: activation of G-protein-coupled receptors (GPCRs) and inhibition of histone deacetylases (HDACs) (63, 64). SCFAs function as cognate ligands for GPCRs—namely GPR41, GPR43, and GPR109A—expressed on intestinal epithelial, adipose, lymphoid, and immune cells, thereby initiating downstream signaling cascades (65, 66). Engagement of GPCRs by SCFAs promotes glucagon-like peptide-1 (GLP-1) secretion, enhances satiety and insulin sensitivity, and improves glucose homeostasis while concurrently suppressing IL-6 and IL-8 production, thus exerting anti-inflammatory effects (63, 67). HDACs are a family of enzymes that remove acetyl moieties from both histone and non-histone proteins, thereby compacting chromatin structure and repressing gene transcription (68). HDACs are indispensable for hepatic glucose homeostasis; their selective suppression lowers fasting glycaemia and ameliorates glucose tolerance (69). SCFAs act as endogenous HDAC inhibitors, and their inhibitory efficacy is concentration-dependent. The underlying mechanisms appear to involve both direct intracellular uptake via specific transporters and indirect suppression mediated through GPCR activation (70).

Bile acids are steroid-derived acids synthesized from hepatic cholesterol and serve as pivotal regulators of lipid digestion and absorption. Their bidirectional interactions with the gut microbiota have been causally linked to the risk of diabetes mellitus (71). Bile acids contribute to metabolic dysregulation through three primary mechanisms: (i) modulation of microbial community structure and function, (ii) intracellular or organ-level accumulation of bile acids, and (iii) perturbation of microbiota-dependent bile-acid signaling (72). The impact of bile acids on microbial composition can be exerted either directly through membrane-disrupting physicochemical effects or indirectly through activation of the nuclear receptor FXR (73). Reciprocally, intestinal microbes enzymatically modify bile-acid pools through deconjugation and dehydroxylation, thereby altering their relative abundance and signaling capacity. For example, enhanced 7α-dehydroxylation by specific bacterial taxa increases the proportion of secondary bile acids, leading to their accumulation within the gut lumen. Excessive luminal bile-acid concentrations can stimulate intestinal immune cells to release proinflammatory cytokines (e.g., TNF-α, IL-6, IL-1β), thereby fostering chronic low-grade inflammation implicated in type 2 diabetes mellitus (T2DM) (74, 75). The principal receptors mediating bile-acid signaling are the farnesoid X receptor (FXR) and the G-protein-coupled receptor TGR5. FXR activation represses hepatic gluconeogenesis by down-regulating the transcription of phosphoenolpyruvate carboxykinase (PEPCK) and glucose-6-phosphatase (G6Pase), thereby ameliorating diet-induced hyperinsulinemia and hyperglycemia (76). In contrast, TGR5 activation promotes GLP-1 secretion, enhances pancreatic β-cell function, augments insulin release, and improves glucose tolerance (77).

Endotoxin is a lipopolysaccharide (LPS) component of the cell wall of Gram-negative bacteria, which is only released in small amounts in the normal gut microbiota. LPS is a key trigger for metabolic diseases and is linked to obesity and insulin resistance (78). A high-fat diet increases the number of intestinal Gram-negative bacteria and the level of plasma LPS. Continuous subcutaneous injections of LPS can cause the same effects as a high-fat diet, including impaired fasting blood glucose, hyperinsulinemia, and increased body weight. CD14 mutant mice (LPS receptor inactivation) delay the onset of insulin resistance and obesity under a high-fat diet and completely block hepatic steatosis (78). Qin J et al. (47) found that the abundance of LPS produced by gut microbiota was positively correlated with fasting blood glucose and HOMA-IR (an assessment indicator of insulin resistance) when performing metagenomic analysis on patients with T2DM. When the number of Gram-negative bacteria in the intestine increases or intestinal permeability changes, the concentration of LPS in serum increases, which may induce endotoxemia and insulin resistance (79). Toll-like receptors (TLRs) can recognize pathogen-associated molecular patterns (PAMPs) from the microbiome and are mainly present on the surface of immune cells (80). LPS binds to the TLR4 receptor, activates the NF-κB pathway, initiates the transcription of proinflammatory genes such as IL-6, IL-1, and TNF-α, causes an inflammatory response, and leads to the occurrence of insulin resistance (81).

Intestinal immune–metabolic homeostasis is orchestrated by the tripartite interplay of SCFAs, BAs, and LPS. SCFAs reinforce epithelial barrier integrity, suppress NF-κB-driven inflammation, counteract LPS translocation and toxicity, and fine-tune bile-acid metabolism via FXR signaling (82). Conversely, BAs sculpt the microbial community through direct antimicrobial activity, thereby modulating SCFAs production (72). LPS perturbs this equilibrium by triggering TLR4-mediated pro-inflammatory cascades (83). Precise spatiotemporal balance among these three metabolite classes is therefore indispensable for maintaining gut microbiota stability and host health.

## 4 The mechanism of *Dendrobium officinale* polysaccharides regulating gut microbiota to improve diabetes mellitus

**Figure 2:**
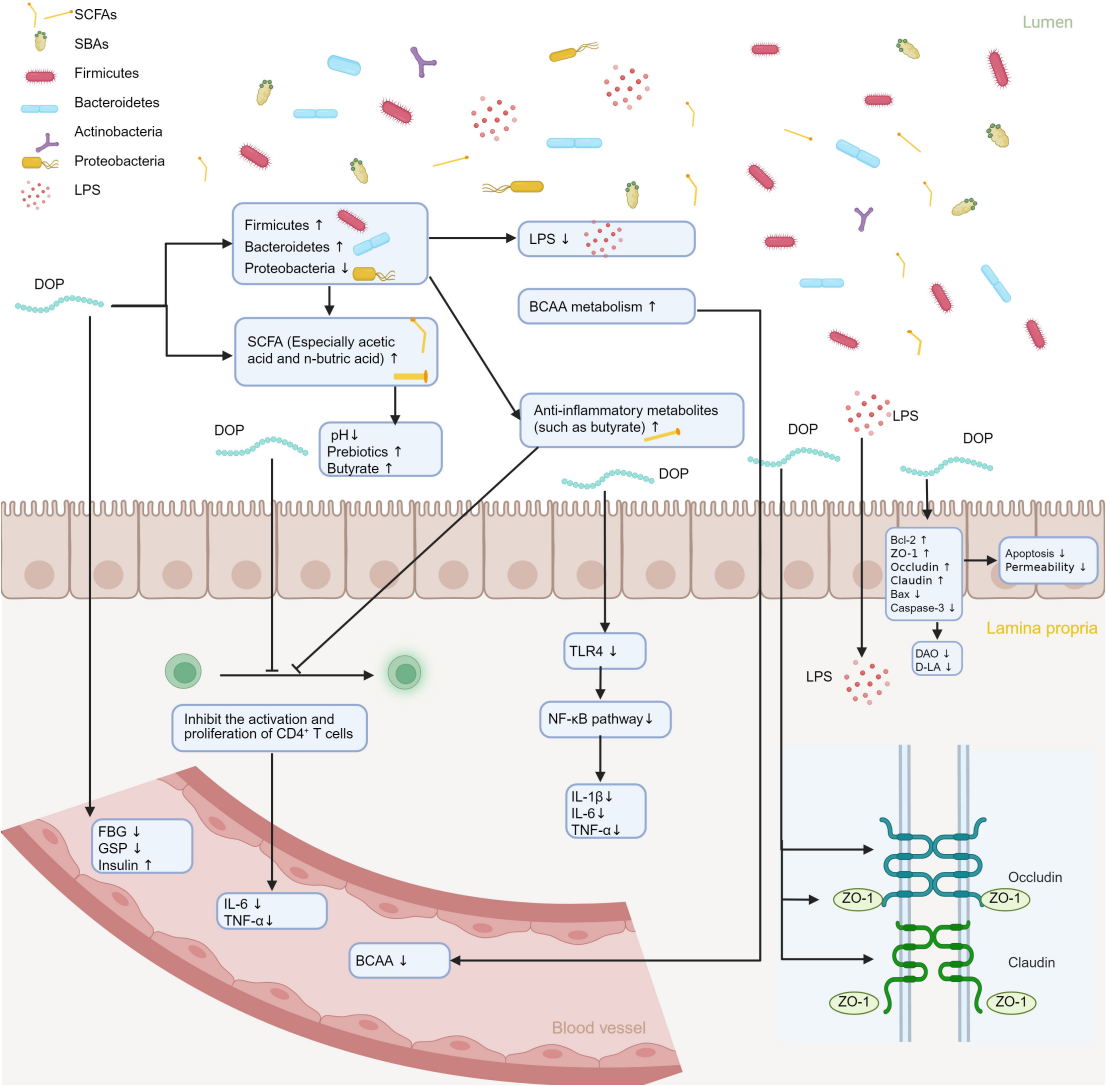
Illustration of the mechanism by which DOPs ameliorates diabetes mellitus via modulation of the gut microbiota. DOPs reshapes microbial composition, thereby altering the profile of microbial metabolites. These changes upregulate tight-junction proteins ZO-1, Occludin, and Claudin, restoring intestinal barrier integrity, while concurrently downregulating pro-inflammatory cytokines IL-1β, IL-6, and TNF-α. Consequently, immune homeostasis is re-established and the metabolic milieu is improved.

Imbalance in gut microbiota is closely correlated with the occurrence and development of diabetes mellitus. Changes in the gut microbiota of diabetic patients can worsen metabolic disorders in the human body. As a traditional Chinese herbal medicine, *D. officinale* has been used to regulate blood sugar and improve metabolism for thousands of years. Modern pharmacological studies have also shown that DOPs are effective in preventing and treating diabetes mellitus (35). Due to its indigestible characteristics, it is suggested that DOPs improve diabetes mellitus by regulating gut microbiota. Chen et al. (84) established a conventional type 2 diabetes mellitus (T2DM) mouse model via high-fat diet feeding combined with streptozotocin injection, and subsequently generated a pseudo-germ-free model by continuous administration of a broad-spectrum antibiotic cocktail in drinking water. The authors demonstrated that the glucolipid-metabolic benefits, suppression of LPS translocation, and anti-inflammatory effects elicited by DOPs are strictly dependent on an intact gut microbiota. As such, DOPs can ameliorate diabetes mellitus via modulation of the gut microbiota (**Figure 2**).

### 4.1 *Dendrobium officinale* polysaccharides regulate the relative abundance of gut microbiota

The bioavailability of DOPs is poor, and their complex polysaccharide structure makes them difficult to digest. It interacts with the gut microbiota in the distal intestine to exert its function (85). An *in vitro* fermentation study on DOPs showed that after 48 hours of co-culture fermentation, 63.88% of the total carbohydrates in the DOPs treatment group were consumed. The concentrations of mannose, glucose and galactose were reduced, and the total SCFA production was significantly increased, indicating that DOPs can be degraded into monosaccharides and utilized by the gut microbiota (34). DOPs have a regulatory effect on the relative abundance of gut microbiota, can regulate the proportion of various types of flora, reduce LPS-producing bacteria (such as *Helicobacter*), enhance the production of short-chain fatty acids, repair the intestinal barrier, and improve metabolic diseases (84). Animal experiments have shown that DOPs significantly increase the abundance of *Firmicutes* and *Bacteroidetes*, while inhibiting *Proteobacteria*, and selectively amplify probiotics (such as *Bifidobacterium*, *Lactobacillus*, and *Allobaculum*) by more than 94% (84). Another study established a T2DM mouse model by high-fat diet combined with streptozotocin and showed that the WDOP1 treatment group and the metformin treatment group showed similar effects. Both could significantly reduce fasting blood glucose, serum insulin levels, and HbA1c levels of T2DM mice, and improve glucose intolerance and insulin resistance (35). In terms of gut microbiota, the WDOE/WDOP1 treatment group reversed the gut microbiota imbalance of T2DM mice, normalized the ratio of *Firmicutes*/*Bacteroidetes*, and reduced the abundance of harmful bacteria (such as *Enterococcus casseliflavus* and *Eubacterium plexicaudatum*) *(* 35).

### 4.2 *Dendrobium officinale* polysaccharides repair intestinal barrier damage

**Figure 3:**
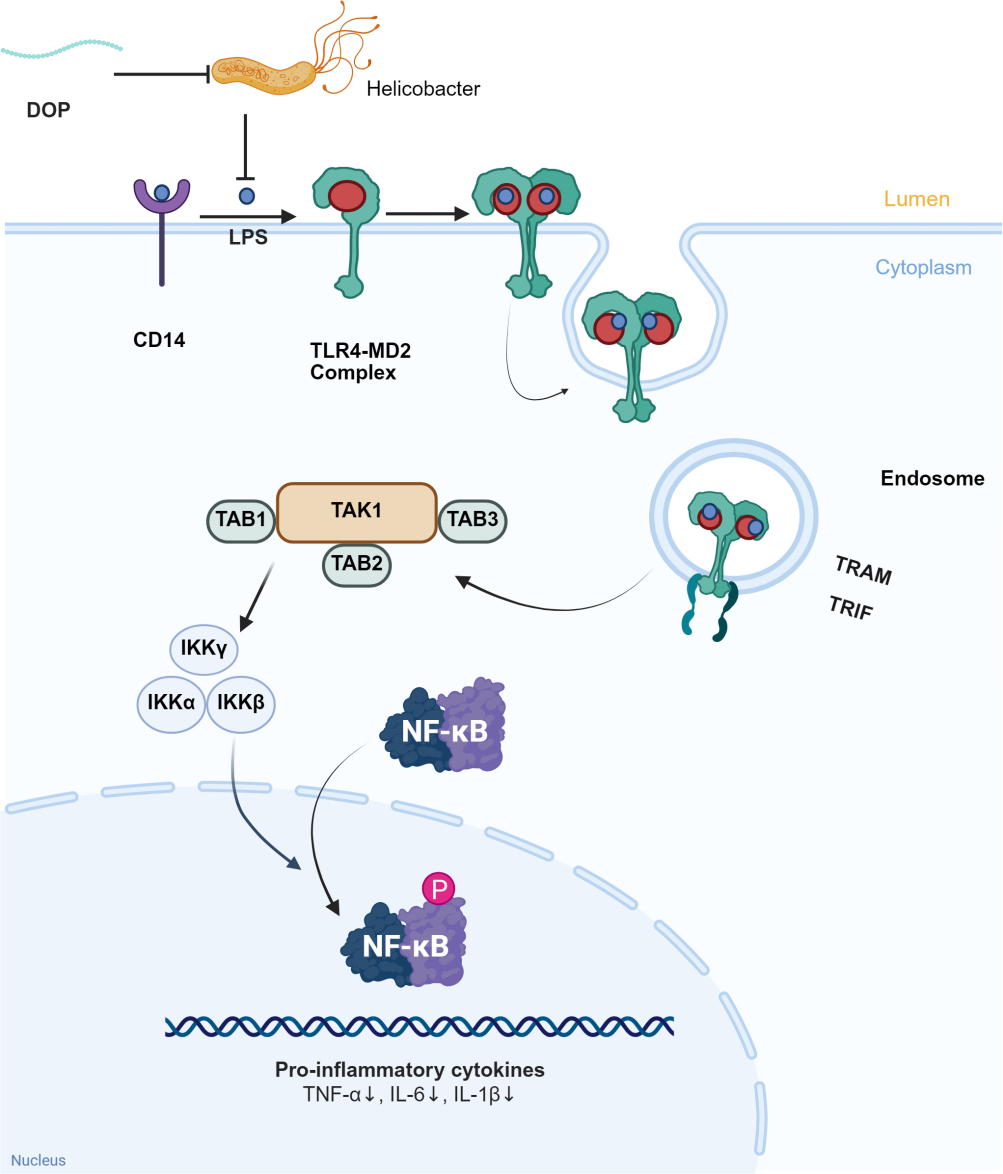
DOPs improve LPS leakage and inflammation levels through gut microbiota. LPS binds to CD14 and TLR4-MD2 complexes in the intestinal lumen, activating downstream signaling molecules TAK1, TAB1, TAB2, and TAB3, which in turn activate IKKα, IKKβ, IKKγ, and further activate NF-κB, exacerbating the inflammatory response. DOPs reduce inflammation by inhibiting *Helicobacter pylori*, decreasing the production and leakage of LPS, and inhibiting the formation of the TLR4-MD2 complex.

Intestinal barrier functions as the defense system which is composed of the intestinal mucosal barrier and intestinal-associated lymphoid tissue. The intestinal epithelium is the key barrier for the body to resist endogenous and exogenous harmful substances. The integrity of the barrier is vital in the stability of the intestinal microenvironment (85, 86). It is well known that increased intestinal permeability leads to bacterial translocation and lipopolysaccharide penetration, causing metabolic endotoxemia. This is associated with autoimmune reactions, chronic inflammation, and promotes the occurrence and development of T1DM and T2DM (87, 88). As shown in **Figure 3**, DOPs inhibit the expression of TLR4 and its downstream signaling molecules TRAM and TRIF by reducing LPS, thereby reducing the phosphorylation of IKKβ and NF-κB p65 and blocking the activation of the NF-κB pathway (84). Importantly, DOPs have been shown to be able to repair intestinal barrier damage. DOPs can reduce intestinal permeability by upregulating the expression of intestinal tight junction proteins (ZO-1, occludin) and Bcl-2 proteins. It also downregulates the expression of Bax and caspase-3 proteins, enhancing the tight junctions between intestinal cells, and reducing intestinal epithelial cell apoptosis (89). In mice with T2DM induced by a high-fat diet combined with streptozotocin, polysaccharides from *D. officinale* upregulated the expression of tight junction proteins ZO-1, Occludin, and Claudin-1. This reduced the levels of intestinal permeability indicators DAO and D-LA, inhibited LPS leakage, inflammation, and oxidative stress, and alleviated insulin resistance (90).

### 4.3 Immunomodulatory effects of polysaccharides from *Dendrobium officinale*

The three polysaccharides of *D. officinale* play a role in immunomodulation, and their impact on immunomodulation may be connected to gut microbiota. Inflammatory factors are related to the advancement of diabetes mellitus and related metabolic disorders and can hinder the function of the insulin signaling pathway, affecting human metabolism. The NF-κB signaling pathway is linked to metabolic diseases. As it disrupts insulin signaling by controlling the expression of proinflammatory cytokines (such as TNF-α, IL-1β, and IL-6), resulting in insulin resistance (91). Research indicates that following DOW-5B treatment, there were significant changes in the levels of IL-10 and TNF-α levels in mouse serum, with a notable increase in IgM levels (92). Polysaccharides DOPs, isolated from the leaves of *D. officinale*, markedly downregulate the expression of IL-1β, IL-6, and TNF-α by inhibiting the TLR4/NF-κB/NLRP3 signaling axis (93). Another animal study demonstrated that DOPs treatment can decrease the levels of two inflammatory cytokines, IL-6 and TNF-α, in serum, and resulted in a decrease in the weight of mice in the DOPs treatment group, possibly due to DOPs inhibiting the activation and proliferation of CD4+ T cells (94). The immune regulatory pathway of DOPs is associated with alterations in gut microbiota composition. DOPs significantly increased the proportion of beneficial bacteria in the intestine (such as *Bifidobacterium*, *Lactobacillus*, etc.), while reducing harmful bacteria in the intestine, increasing anti-inflammatory metabolites like butyrate in the intestine, thereby restraining the overactivation of CD4+ Th cells, decreasing inflammatory factors such as TNF-α/IL-6, and achieving a flora-immune synergistic regulation (94).

### 4.4 *Dendrobium officinale* polysaccharide improves the metabolic environment

**Figure 4:**
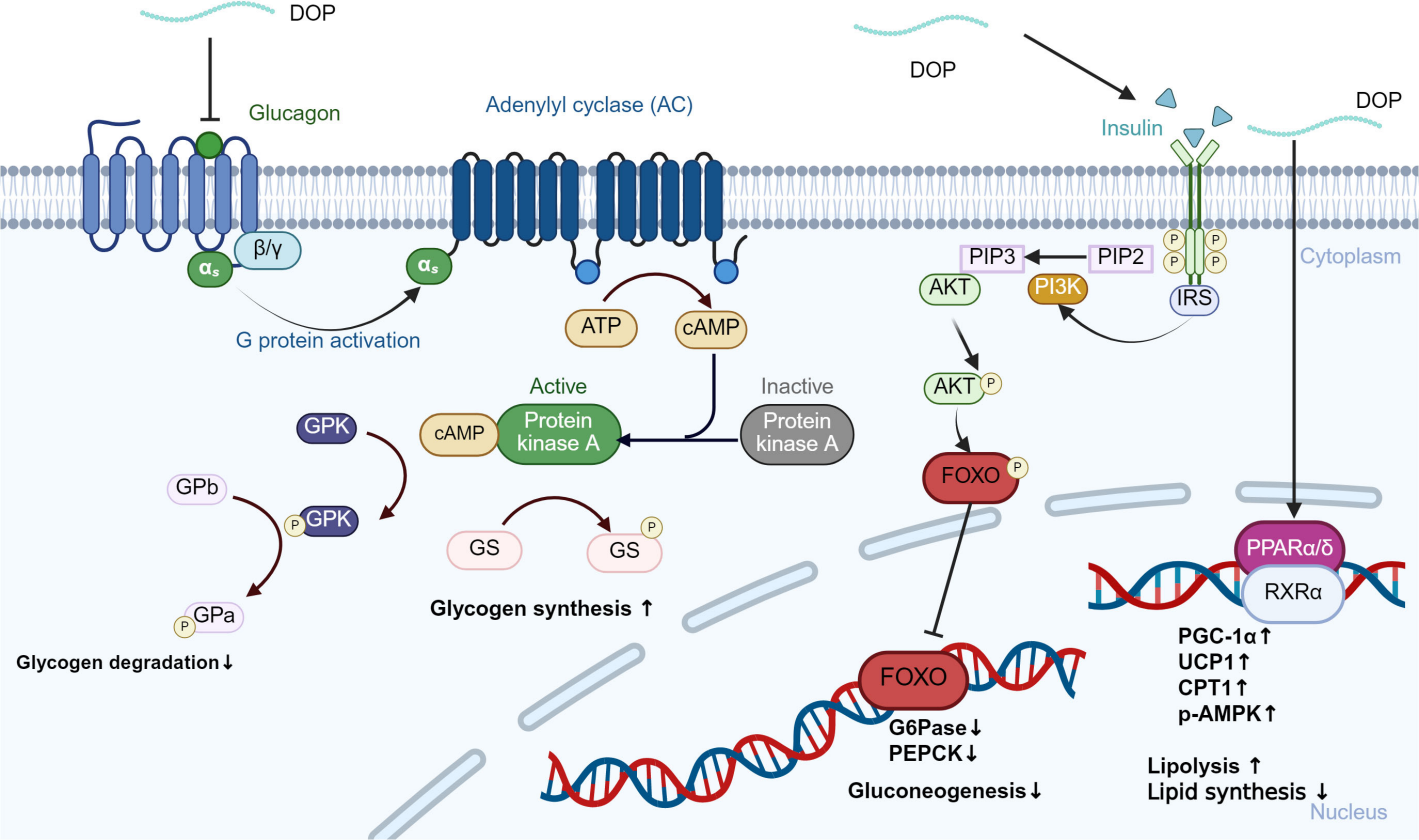
DOPs-mediated regulation of hepatic glucagon and insulin signaling pathways. The binding of glucagon to the glucagon receptor (GCGR) activates adenylyl cyclase (AC), leading to increased cAMP levels and subsequent activation of protein kinase A (PKA). This cascade promotes glycogen degradation by phosphorylating glycogen phosphorylase (GP) and inhibiting glycogen synthase (GS). Insulin and DOPs activate the PI3K/Akt pathway, which phosphorylates and inactivates FoxO1, thereby reducing the expression of gluconeogenic enzymes PEPCK and G6Pase.

Long-term high-fat, high-calorie diets inhibit the activity of liver lipoprotein lipase (LPL), hinder lipoprotein catabolism, increase blood triglyceride (TG) and low-density lipoprotein cholesterol (LDL-C) levels, lead to metabolic disorders, and increase the risk of diabetes mellitus (95, 96). Oral administration of DOPs can increase serum insulin and reduce fasting blood glucose (FBG) and glycosylated serum protein (GSP) levels in alloxan-induced diabetic mice, which may be related to its ability to repair damaged pancreatic tissue and improve insulin secretion (97). *In vitro* experiments have also shown that DOPs can improve obesity-related insulin resistance and abnormal lipid metabolism, and its effects are closely related to PPAR-γ (98). DOPs can lower blood glucose by suppressing hepatic gluconeogenesis via inhibition of the glucagon-cAMP-PKA and Akt/FoxO1 pathways while enhancing glycogen synthesis and stability (99). Furthermore, concurrent activation of the PPAR-RXR axis increases fatty acid oxidation and insulin sensitivity, resulting in a sustained reduction of fasting glucose and improved glucose tolerance (100). Together, these data suggest that DOPs can affect glucose metabolism (**Figure 4**). DOPs can also significantly improve amino acid metabolism disorders in diabetic rats. In animal experiments, it was found that the ability of diabetic rats to metabolize branched-chain amino acids (BCAA) weakened, but the content in their blood was significantly increased. At the same time, the number of genes related to BCAA synthesis in the gut microbiota increased. DOPs treatment reduced the abundance of genes related to BCAA biosynthesis and improved BCAA metabolism (101). The gut microbiota participates in carbohydrate metabolism and nutrient absorption, producing products such as short-chain fatty acids SCFAs. Imbalance in the flora can interfere with blood sugar metabolism and increase the risk of diabetes. DOPs can significantly increase the content of total SCFAs in the colon of mice, especially acetic acid and butyric acid, and reduce the pH value of the colon environment, which is conducive to the production of prebiotics and butyrate, thereby regulating the balance of the flora and improving intestinal health (102).

## 5 Conclusion

As a metabolic disease, diabetes mellitus is linked to human metabolic disorders and gut microbiota imbalance. In recent years, improving diabetes mellitus and its complications through gut microbiota has become a research hotspot, and diabetes mellitus therapy based on gut microbiota has become a new treatment idea after traditional therapy. As a traditional Chinese medicine, the effective active ingredient of DOPs has shown potential pharmacological effects in the treatment of diabetes mellitus. By regulating the relative abundance of gut microbiota, repairing intestinal barrier damage, regulating immune function, and improving the metabolic environment, it neutralizes the imbalance of gut microbiota, weakens the inflammatory state, improves metabolic disorders, and is beneficial to the treatment of diabetes mellitus. Natural compounds represented by DOPs are expected to provide new intervention strategies for the treatment of diabetes-related diseases. In the future, DOPs can be further studied as a prebiotic and combined with probiotics in adjuvant therapy or dietary intervention for diabetic patients, especially for patients with prediabetes. In clinical practice, a new “bacteria-drug synergy” intervention strategy is provided for diabetic patients, targeting gut microbiota and performing compatibility quantification based on the patient’s baseline flora characteristics. However, it is worth noting that although the prebiotic potential of DOPs is supported by animal studies and *in vitro* data, there are currently no registered or published human clinical trials testing their effects on gut microbiota or metabolic outcomes. Therefore, the optimal dose-response relationship, intervention duration, and long-term safety of DOPs as candidate prebiotics have not yet been determined. In addition, the development of functional foods of DOPs can be promoted to provide low-dose, long-term safety solutions for people with sub-health and chronic diseases, reducing drug dependence and medical expenses. However, some problems still need to be solved to achieve its widespread clinical application. The biological activities of DOPs are related to their chemical structure and molecular weight. The relationship between structure and function needs to be clarified. The optimal extraction method and dosage need to correspond to its applicable population and efficacy. In addition, the mechanism of DOPs acting on gut microbiota is not yet completely clear. DOPs lack large-scale double-blind clinical trials to verify the results of animal experiments and *in vitro* experiments to ensure their safety and effectiveness in humans. Efforts can also be put into the combined application effect of DOPs and other anti-diabetic drugs to provide a new perspective for the clinical treatment of diabetes mellitus.
